# The Tissue-Specific Rep8/UBXD6 Tethers p97 to the Endoplasmic Reticulum Membrane for Degradation of Misfolded Proteins

**DOI:** 10.1371/journal.pone.0025061

**Published:** 2011-09-15

**Authors:** Louise Madsen, Franziska Kriegenburg, Andrea Vala, Diana Best, Søren Prag, Kay Hofmann, Michael Seeger, Ian R. Adams, Rasmus Hartmann-Petersen

**Affiliations:** 1 Department of Biology, University of Copenhagen, Copenhagen, Denmark; 2 MRC Human Genetics Unit, Institute of Genetics and Molecular Medicine, Western General Hospital, Edinburgh, Scotland; 3 Instituto de Medicina Molecular, Faculdade de Medicina da Universidade de Lisboa, Lisboa, Portugal; 4 Bioinformatics Department, Miltenyi Biotec GmbH, Bergisch-Gladbach, Germany; 5 Institut für Biochemie, Charité – Universitätsmedizin Berlin, Berlin, Germany; University of South Florida College of Medicine, United States of America

## Abstract

The protein known as p97 or VCP in mammals and Cdc48 in yeast is a versatile ATPase complex involved in several biological functions including membrane fusion, protein folding, and activation of membrane-bound transcription factors. In addition, p97 plays a central role in degradation of misfolded secretory proteins via the ER-associated degradation pathway. This functional diversity of p97 depends on its association with various cofactors, and to further our understanding of p97 function it is important that these cofactors are identified and analyzed. Here, we isolate and characterize the human protein named Rep8 or Ubxd6 as a new cofactor of p97. Mouse Rep8 is highly tissue-specific and abundant in gonads. In testes, Rep8 is expressed in post-meiotic round spermatids, whereas in ovaries Rep8 is expressed in granulosa cells. Rep8 associates directly with p97 via its UBX domain. We show that Rep8 is a transmembrane protein that localizes to the ER membrane with its UBX domain facing the cytoplasm. Knock-down of Rep8 expression in human cells leads to a decreased association of p97 with the ER membrane and concomitantly a retarded degradation of misfolded ER-derived proteasome substrates. Thus, Rep8 tethers p97 to the ER membrane for efficient ER-associated degradation.

## Introduction

When correct folding of proteins or assembly of oligomeric proteins in the endoplasmic reticulum (ER) is disturbed, misfolded or unassembled proteins accumulate inside the ER lumen. If such proteins are allowed to linger, they may form insoluble aggregates and thus pose a serious threat to the cell. Normally, cells rapidly channel misfolded proteins back into the cytoplasm for degradation by the ubiquitin-proteasome system [1]–[7]. The recognition of the misfolded proteins in the ER lumen and subsequent transport to the proteasome is termed ER quality control [3], [5] and ER-associated degradation (ERAD) [1]–[5], respectively. Extraction of misfolded proteins from the ER relies on the ring-shaped homohexameric ATPase known as p97 or VCP (valosin-containing protein) in mammals and Cdc48 in yeast [8]–[12].

The p97 ATPase is a member of the AAA (ATPase associated with various activities) family of ATPases [13]. The monomer is a phylogenetically highly conserved and essential protein that carries two AAA modules, called D1 and D2. These link coordinated ATP-hydrolysis to conformational changes of the hexameric complex [14], in a fashion similar to other AAA-type ATPases [15]. The ATP-powered conformational changes allow p97 to drive the disassembly of protein complexes and segregate proteins from their binding partners [16], [17]. This catalytic activity of p97, termed the “segregase” activity [10], [18], is probably restricted to ubiquitylated proteins and is important for a number of cellular pathways, including membrane fusion [19], protein degradation [11], [20], and transcription factor maturation [18], [21]. Although p97 may bind ubiquitylated proteins directly [18], a series of p97 cofactors recruit and/or process substrates [22]–[24]. The functions of these cofactors are diverse, and each probably directs p97 activity to a particular cell function. For instance, the UBX domain protein, p47, directs p97 to functions in membrane fusion [19], while another UBX domain protein, Ubxd7, targets the HIF1α transcription factor for degradation [24]. With only one exception, Ubxd1 [25], [26], all characterized UBX-domain proteins associate directly with p97 via their UBX domains [24], [27], [28].

During ERAD, p97 also relies heavily on cofactors. Initially, p97 associates with the cytosolic side of the ER membrane by interacting with transmembrane p97 cofactors such as VIMP and the UBX protein, Erasin [29], [30]. These cofactors are tightly associated with numerous other proteins that probably form a channel through which the misfolded proteins traverse from the ER lumen to the cytosol. These retrotranslocation complexes also contain E2 ubiquitin-conjugating enzymes and E3 ubiquitin-protein ligases that ubiquitylate the substrates as they emerge [2], [4]. The heterodimeric cofactor Ufd1-Npl4 may simultaneously interact with p97 and ubiquitylated substrates [31], [32] and assist p97 with substrate recruitment in ERAD [10] and other degradation pathways [33]. Then, peptide:*N*-glycanase associates with p97 and removes glycans from glycosylated ERAD substrates [34] before the substrate is finally transferred to the 26S proteasome and degraded.

To broaden our understanding of p97 it is critical to obtain a more detailed knowledge of its various cofactors, of which at least 20 have been found so far [22], [23]. Certainly, some functions of p97 are likely to be specific to certain cells or tissues. For instance, cells that secrete large amounts of protein, such as plasma and liver cells, probably have a highly active ERAD system and ERAD-relevant p97 cofactors may therefore be more abundant in such cells. However, so far no cell- or tissue specific p97 cofactors have been isolated or characterized. Here, we describe the tissue-specific human protein Rep8 (also known as Ubxd6), a previously cloned [35], but otherwise uncharacterized UBX protein present in higher eukaryotes. We found that Rep8 shares some similarity with the transmembrane p97 cofactor called VIMP [29]. Accordingly, we found that Rep8 is a transmembrane protein, localized in the ER membrane. Rep8 associates with p97 via its cytoplasmic UBX domain to the N-domain of p97. In agreement with a previous report [35], we find that Rep8 is almost exclusively expressed in gonads. In the testis we find Rep8 expression in post-meiotic round spermatids. In ovaries Rep8 is expressed in granulosa cells. Knock-down of Rep8 expression results in delayed clearance of model ERAD substrates and reduced amounts of p97 associated with the ER membrane. Thus, like VIMP, Rep8 probably facilitates ERAD by tethering p97 to the ER membrane.

## Materials and Methods

### Buffers

The buffers were: Buffer A, 25 mM Tris/HCl pH 7.5, 2 mM MgCl_2_, 2 mM ATP, 50 mM NaCl, 1 mM DTT, 10% (v/v) glycerol, 0.1% (v/v) Triton X-100. Buffer B, 33 mM Hepes pH 7.3, 150 mM potassium acetate, 10% (v/v) glycerol, 1% (w/v) DeoxyBigChap (Fluka). Buffer C, 33 mM Hepes pH 7.3, 150 mM potassium acetate, 10% (v/v) glycerol, 0.2% (w/v) DeoxyBigChap (Fluka), 1 mg/mL BSA. Buffer D, 20 mM Hepes pH 7.5, 0.25 M sucrose, 1 mM DTT. Buffer E, 25 mM Tris/HCl pH 8.0, 500 mM NaCl, 1 mM DTT.

### Plasmids and expression

For expression of recombinant Rep8, full-length cDNA and various truncations encoding human Rep8 were transferred to the appropriate Gateway destination vectors (Invitrogen). The expression constructs for mouse p97 were kindly provided by Dr. Hemmo H. Meyer (Zurich, Switzerland). The proteins were expressed in *E. coli* BL21*(DE3) (Invitrogen) and purified by standard methods.

The plasmid, used for expressing ER-targeted RFP, was generously supplied by Dr. Ulrike Kutay (Zurich, Switzerland).

### Yeast two-hybrid screening

Yeast two-hybrid screening, using full length human p97, was performed on a HeLa cell cDNA library (Invitrogen) using the ProQuest yeast two-hybrid system (Invitrogen) according to the protocol, supplied by the manufacturer.

### Cell culture

MelJuSo cells, stably transfected to express CD3δ-YFP and ubiquitin^G76V^-YFP, were generously supplied by Dr. Nico P. Dantuma (Stockholm, Sweden). HeLa cells, stably transfected to express HA-tagged TCRα, were kindly supplied by Dr. Cezary Wójcik (Evansville, Indiana). These cells and HeLa cells that were stably transfected to express Hrd1 with a biotin targeting sequence [25], were maintained in Dulbecco's modified Eagle's minimal essential medium (DMEM) supplemented with 10% newborn- or fetal-calf serum (Invitrogen) at 37°C in a humidified atmosphere containing 5% CO_2_.

### Electrophoresis and blotting

Proteins were separated on 7 cm×8 cm 12.5% acrylamide gels. Proteins were transferred to BA83 (Schleicher & Schuell) nitrocellulose membranes by semi-dry blotting and probed with antibodies as indicated. Densitometry was performed using UnScanIt v.6.1 software (Silk Scientific).

### Purification of p97 from red blood cells

Untagged p97 was purified from outdated human red blood cells by following a protocol for purification of 26S proteasomes [36]. After the final chromatographic step, fractions not containing 26S proteasomes were analyzed by dot blotting for the presence of p97. The p97-containing fractions were pooled and found by SDS-PAGE to contain pure p97.

### Antibodies

Antibodies to human Rep8 were raised in rabbits by immunization with purified GST-tagged Rep8 residues 57–270, encompassing the entire cytoplasmic domain of Rep8. The antibodies to p97 have been described previously [37]. Antibodies to TMX3, calnexin, and ERp57 were generously supplied by Dr. Lars Ellgaard. Antibodies specific for proteasome subunits were from Enzo Life Sciences. Anti-pentaHis antibodies were purchased from Qiagen. The anti-GST, anti-GFP, anti-HA and anti-β-actin were purchased from Sigma. Peroxidase conjugated streptavidin was purchased from Dako.

### Assays

The concentration of cell protein was determined using BCA (Pierce) or Bradford (Pierce) assays with BSA as a standard. Concentrations of purified recombinant proteins were determined from A_280nm_.

### Transfection

Small interfering RNAs (HP GenomeWide siRNA SI04350934 and SI04323347), specific for human Rep8, were purchased from Qiagen. The siCONTROL siRNA#1 (Dharmacon) was used as an unspecific control. Exponentially growing MelJuSo or HeLa cells were washed in PBS and incubated for 24 h with 100 nM siRNA and 0.4% Dharmafect in DMEM supplemented with 1% calf serum. The medium was then changed to DMEM with 10% serum. The cultures were used after another 4 days.

### Co-precipitations

Transformed *E. coli* BL21*(DE3) cells, expressing tagged protein, were lysed by sonication in one volume of buffer A. The extracts were cleared by centrifugation (12000 g, 30 min) and the fusion protein was purified. For precipitation experiments with HeLa cells stably transfected to express Hrd1 with a biotin tag, the cells were suspended in 4 volumes of buffer B and incubated with gentle agitation for 30 min. Cleared extracts were prepared by centrifugation as above. Aliquots of extracts were incubated at 4°C for 4 h with 20 µL glutathione Sepharose beads (GE Healthcare) loaded with GST or the GST-tagged proteins. The beads were washed in 3×15 mL of buffer A for recombinant proteins or buffer C for HeLa cell extracts. The precipitation experiments with purified p97 were performed in buffer A using about 3 µg p97 per reaction. Bound proteins were analyzed by SDS-PAGE and immunoblotting.

### Differential centrifugation

Differential centrifugation for subcellular localization of Rep8 was performed essentially as described [38]. To quantify the amount of p97 associated with cell membranes, dilution series of the appropriate fractions were quantified by SDS-PAGE and immunoblotting.

### Membrane topology

The orientation of Rep8 in the ER membrane was determined by Proteinase K and Triton X-100 treatment of microsomes. Briefly, HeLa cells were harvested and lysed in buffer D by passing through a 27 gauge needle. Unbroken cells were removed by centrifugation (300 g, 3 min) and the membrane fraction isolated by centrifugation (100000 g, 1 h). The membranes were resuspended in buffer E. Aliquots of the membrane fraction were treated on ice with 1 mg/mL proteinase K (Sigma) and/or 1% Triton X-100 for 1 h. PMSF was then added to 5 mM before the samples were subjected to TCA precipitation. The precipitates were washed with ice cold acetone, resuspended in SDS sample buffer and resolved by SDS-PAGE and immunoblotting.

### Pulse-chase experiments

The stability of the model substrates was followed by pulse-chase analysis of transfectants stably expressing the substrate, as described previously [39].

### Fluorescence microscopy

For fluorescence microscopy, HeLa cells were transiently co-transfected with plasmids for expressing Rep8 with a C-terminal GFP-tag and RFP modified to contain a signal sequence and the ER retention sequence, KDEL. About 48 h after transfection, the cells were fixed with 4% formaldehyde in PBS and mounted for confocal microscopy as described previously [25].

### In situ hybridization

Bases 1–559 of mouse Rep8 cDNA (Genbank sequence accession BC024492) from an IMAGE full-length cDNA plasmid (ImaGenes) were subcloned into pCMV-SPORT6, and used to generate sense and anti-sense digoxigenin-labelled Rep8 RNA probes by *in vitro* transcription. *In situ* hybridization to Bouin's fixed adult mouse ovary and testis sections was performed as described [40]. Bound probe was visualized with NBT/BCIP substrate (Vector Laboratories) and sections counterstained with nuclear fast red.

### qRT-PCR

Oligo dT-primed cDNA was prepared from testes of prepubertal or adult *Tex19.1^−/−^* and littermate control mice as described [41]. Sequences of primers, used for qRT-PCR, are listed in the supplementary material (Table S1). 20 µL of qPCRs containing 250 nM of each primer and 1× Brilliant II SYBR green master mix (Stratagene) were set up in triplicate and run on a Bio-Rad C1000 thermal cycler equipped with a CFX96 real time system. Primer pairs were validated as amplifying at 95–100% efficiency, and expression levels were calculated relative to β-actin, using the 2^−ΔΔCT^ method. No significant qPCR amplification was detected in control cDNAs generated in the absence of reverse transcriptase (not shown).

### Ethics

Transgenic animals used for the experiments in this study were bred and used under the authority and ethical approval of the UK Home Office (Project Licence PPL60/3785).

## Results

### Rep8 interacts with p97

**Figure 1:**
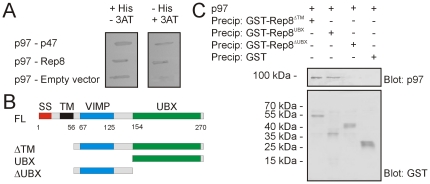
Rep8 interacts with p97 via the UBX domain. (A) Yeast two-hybrid analyses of the p97-Rep8 interaction using the *HIS3* reporter gene. Co-transformation of bait p97 with prey Rep8 supported cell growth under conditions selecting for the interaction (in the absence of histidine and presence of 25 mM 3-aminotriazol (3AT); right panel). The known p97-p47 interaction served as a control. (B) Schematic diagram of the Rep8 domain organization and the various truncations used in the precipitation experiments. (C) Purified p97 was incubated with the indicated, immobilized GST fusion proteins before analysis of bound proteins by SDS-PAGE and blotting using antibodies specific for p97 (top panel) or GST (lower panel).

In a yeast two-hybrid screen of a HeLa cell cDNA library, using human p97 as a bait, the conserved but uncharacterized protein named, Rep8 or Ubxd6 (Swiss-Prot accession: O00124), was isolated (Fig. 1A). The human protein is 73% identical to its murine orthologue and 39% identical to its orthologue in zebrafish (Fig. S1). Database analyses indicate that Rep8 is only found in higher eukaryotes. The primary structure of Rep8 revealed that the protein contains a signal sequence followed by a transmembrane domain, while the C-terminus contains a UBX domain (Fig. 1B and Fig. S1). The UBX domain is regarded as a general p97-interacting domain [24], indicating that Rep8 was a valid target of p97 in the yeast two-hybrid screen. In addition, the R..FPR motif known from other UBX proteins to play a critical role in p97 binding [22] is conserved in Rep8.

By unbiased sequence searching, we also found that Rep8 shows some similarity to the transmembrane p97 cofactor known as SelS or VCP-interacting membrane protein (VIMP) (Fig. 1B and Fig. S1). The VIMP homology in Rep8 is located between the transmembrane region and the UBX domain and matches the major helical part of VIMP [PDB: 2Q2F].

In order to confirm the yeast two-hybrid interaction, GST, GST-tagged Rep8, and GST-tagged truncations of Rep8 (Fig. 1B) were expressed and purified from *E. coli*. To circumvent the likely folding issues of a recombinant transmembrane protein expressed in bacteria, we also deleted the first 56 amino acids encompassing the predicted signal sequence and transmembrane domain (Fig. 1B). The fusion proteins were used in precipitation experiments with p97, purified from human red blood cells. GST-Rep8 precipitated p97, whereas under the same conditions GST did not (Fig. 1C), thus confirming that Rep8 and p97 interact directly. Precipitation using the Rep8 truncations revealed that the C-terminal UBX domain was necessary and sufficient for p97 binding (Fig. 1C).

**Figure 2:**
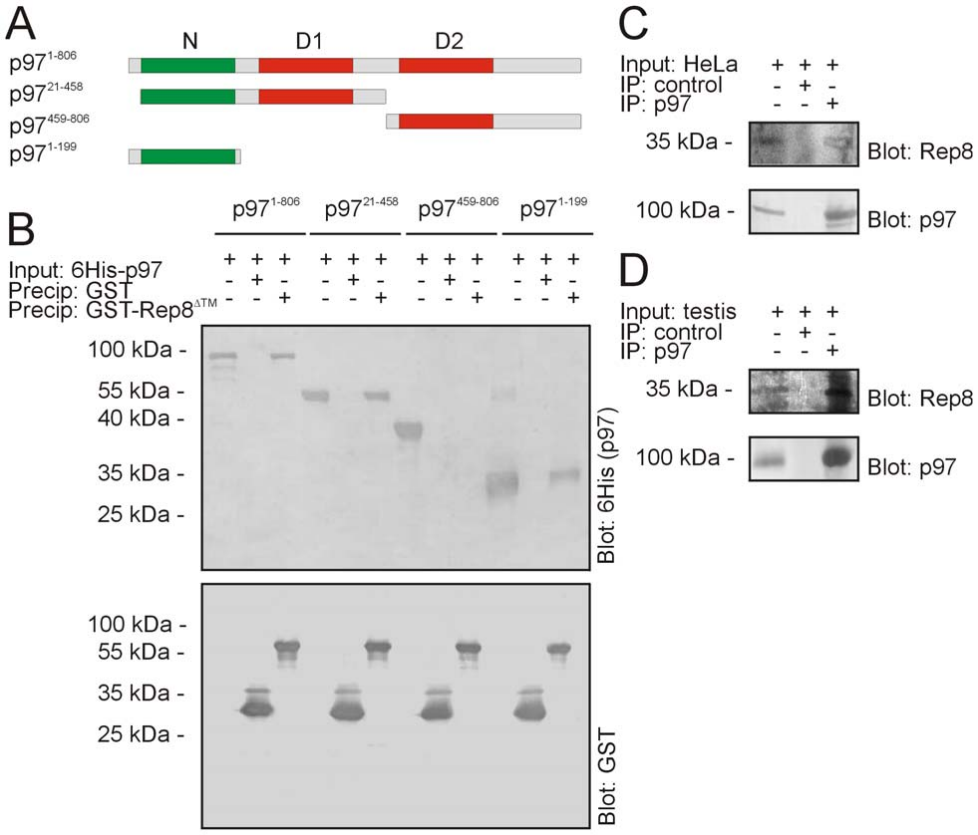
Rep8 interacts with the p97 N-domain. (A) Schematic diagram of the p97 domain organization and the various truncations used in the precipitation experiments. (B) Purified 6His-tagged p97 and p97 truncations were incubated with immobilized GST or GST-tagged Rep8 before analysis of the bound proteins by SDS-PAGE and blotting using antibodies specific for the 6His-tagged p97 proteins (top panel) or GST (lower panel). The p97 input has been included for comparison. (C) Endogenous p97 was immunoprecipitated from HeLa cell lysates. The precipitated material was analyzed by blotting using antibodies to p97 and Rep8. (D) Endogenous p97 was immunoprecipitated from rat testis homogenate. The precipitated material was analyzed by blotting using antibodies to p97 and Rep8.

To more thoroughly analyze the interaction between Rep8 and p97, we sought to map the Rep8 binding site on p97. Most p97-interacting proteins interact with either the N-domain on p97 or a short motif in the C-terminus. In general, UBX domain proteins interact with the N-domain [22]. Accordingly, precipitation experiments with GST-tagged Rep8 and various 6His-tagged p97 truncations (Fig. 2A) revealed that Rep8 interacts with the p97 N-domain (Fig. 2B).

To analyze the interaction between endogenous p97 and Rep8, we raised an antibody to Rep8, and used it to probe p97 immunoprecipitates for Rep8 from HeLa (Fig. 2C) and rat testis lysates (Fig. 2D). Again, we found that Rep8 was bound to p97 (Fig. 2C and 2D), showing that the two proteins are also associated *in vivo*.

### Rep8 is a transmembrane protein localized to the ER membrane

**Figure 3:**
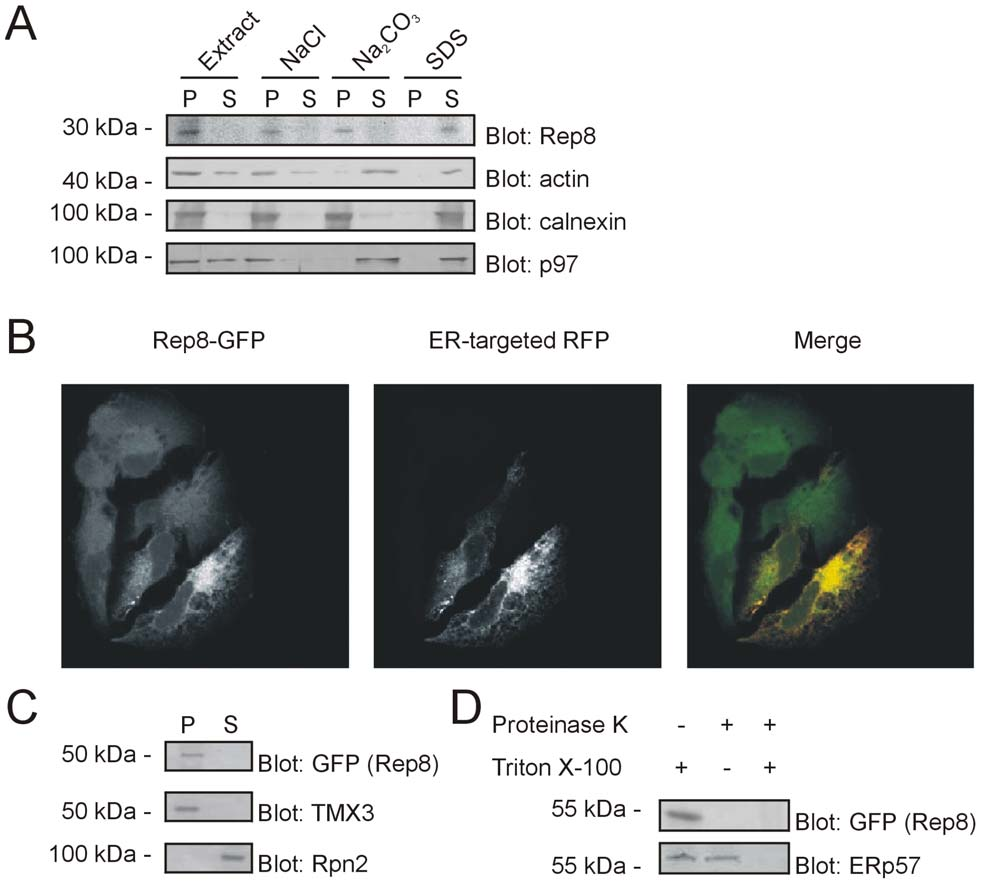
Rep8 localizes to the ER membrane. (A) The insoluble fraction of a HeLa cells lysate was isolated by centrifugation. Pellets were mixed with sucrose, 0.5 M NaCl, 100 mM Na_2_CO_3_ or 0.1% SDS as indicated, and separated by centrifugation into a pellet (P) and supernatant fraction (S) prior to analysis by SDS-PAGE and blotting using antibodies to p97, calnexin, actin and Rep8. To ease comparison, the pellets were resuspended in the same volumes as the supernatant prior to analysis. All Rep8 was insoluble and only released with SDS, but not with sucrose, sodium chloride or sodium carbonate. (B) Confocal micrographs of formaldehyde-fixed HeLa cells transfected to express Rep8 with a C-terminal GFP-tag (left panel) and RFP modified to contain a signal sequence and an ER-retention signal (ER-targeted RFP; middle panel). In the merged image (right panel), the signals overlap (yellow). (C) Differential centrifugation of HeLa cells transfected to express Rep8-GFP as in (A). TMX3 and the proteasome subunit Rpn2 served as controls for a transmembrane and soluble protein, respectively. (D) Microsomes from HeLa cells expressing Rep8 with a C-terminal GFP tag were treated with proteinase K and Triton X-100 as indicated, before they were analyzed by SDS-PAGE and blotting. The C-terminus of Rep8 was detected by an antibody to the GFP-tag. The luminal ER protein, ERp57, served as a control.

Next, we sought to determine the subcellular localization of Rep8. To this end, the antibody to Rep8 was used to analyze HeLa cell components separated by differential centrifugation. As a control, we analyzed the fractions for the peripheral membrane protein p97 and the transmembrane protein calnexin (Fig. 3A). The association of these proteins with membranes were as expected, indicating that the membrane fractionation was successful. We found that all Rep8 was associated with the high-speed pellet and was released when the membranes were treated with detergent but not by washing with sodium chloride or sodium carbonate (Fig. 3A), indicating that Rep8 is indeed, as predicted, a transmembrane protein.

We also analyzed the localization of Rep8 by fluorescence microscopy. Since our antibodies to Rep8 were not suitable for immunofluoresence, HeLa cells were transiently transfected to express full length Rep8 with a C-terminal GFP-tag. The GFP-signal appeared at the ER, and co-localized with RFP that had been modified to contain a signal sequence and an ER retention signal (ER-targeted RFP) (Fig. 3B). Interaction with p97 was not required for the ER localization of Rep8, since a Rep8 truncation, lacking the UBX domain, also localized to the ER (not shown). Differential centrifugation revealed that, like endogenous Rep8, all the Rep8-GFP fusion protein was associated with the microsome pellet (Fig. 3C).

To determine the membrane topology of Rep8, microsomes from transfected cells were treated with proteinase K. While the ER luminal protein ERp57 was resistant to proteinase K treatment (Fig. 3D), the Rep8-GFP signal disappeared completely upon treatment with proteinase K (Fig. 3D), indicating that the C-terminal GFP-tag is oriented towards the cytoplasm. As a control, the microsomes were treated with both detergent and proteinase K, which, as expected, led to degradation of both Rep8 and the luminal ERp57 (Fig. 3D).

### Rep8 is expressed primarily in reproductive tissue

**Figure 4:**
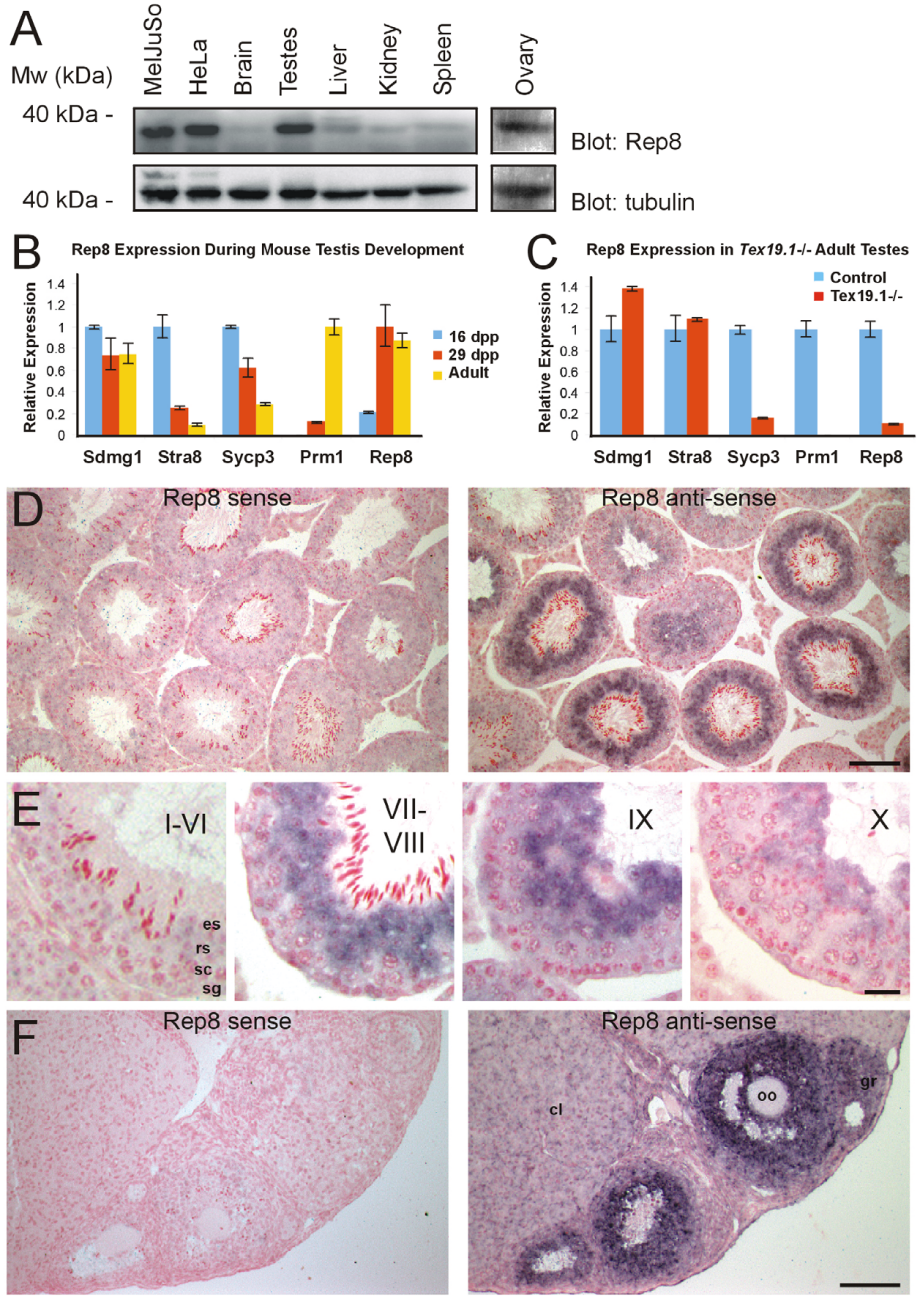
Expression of Rep8. (A) The indicated rat tissues and cell extracts were analyzed by SDS-PAGE and blotting using antibodies specific for Rep8 (upper panel) and tubulin (lower panel). Rep8 was expressed almost exclusively in testes, ovaries and in the cell types used in this study. (B) qRT-PCR for Rep8 expression during mouse testis development. Bars indicate mean expression relative to β-actin, normalized to the maximum expression level for that gene during the developmental time course. Error bars indicate standard errors. (C) qRT-PCR for Rep8 expression in adult *Tex19.1^−/−^* testes. Mean expression levels relative to β-actin were normalized to control adult testes for each gene. Error bars indicate standard errors. (D) *In situ* hybridization of Rep8 to adult mouse testes. Bound sense or anti-sense Rep8 probes were visualized with dark blue/purple precipitate. Sections were counterstained with nuclear fast red. Scale bar 100 µm. (E) Higher magnification images of Rep8 *in situ* hybridization to adult testes. The approximate seminiferous epithelial stage is indicated by roman numerals, and examples of mitotic spermatogonia (sg), meiotic spermatocytes (sc), round spermatids (rs) and elongated spermatids (es) are annotated. Scale bar 20 µm. (F) *In situ* hybridization of Rep8 to adult mouse ovary. Scale bar 100 µm. Granulosa cells (gr), oocytes (oo) and corpora lutea (cl) are indicated.

Previous studies have shown that Rep8 mRNA is primarily present in reproductive tissues [35]. To determine the tissue distribution of Rep8 on the protein level, we separated protein extracts from various rat tissues by SDS-PAGE and probed blots for the presence of Rep8. We found that Rep8 was almost exclusively expressed in testes and ovaries, but also present in the cell lines used here (Fig. 4A). Although both cell types used here are derived from human cancers, searching online databases (e.g. Oncomine, BioGPS) did not reveal any clear correlation between Rep8 expression and cancer (data not shown).

Testes contain a mixture of different somatic cell types and germ cells at various stages of differentiation. In order to investigate which cell type in the testis is responsible for the high expression levels of Rep8, we performed qRT-PCR on developing testes from prepubertal mice undergoing the first wave of spermatogenesis. The most advanced male germ cells in prepubertal testes are undergoing mitotic proliferation at around 6 days post partum (dpp), progressing through the pachytene stage of meiosis at around 16 dpp, differentiating into early round spermatids at around 20 dpp, and starting to elongate at around 30 dpp. The Sdmg1 marker for somatic Sertoli cells [40], the Stra8 marker for mitotic (spermatogonia) and early meiotic (preleptotene spermatocyte) male germ cells [42], the Sycp3 marker for meiosis [43] and the Prm1 marker for round and elongating spermatids [44] were all expressed, as would be expected during this developmental time course (Fig. 4B). Rep8 expression in prepubertal testes increased dramatically between the 16 dpp and 29 dpp time points, coincident with the appearance of late meiotic spermatocytes and post-meiotic round spermatids in the testis (Fig. 4B). Furthermore, adult testes from *Tex19.1^−/−^* mice, which contain all the somatic cell types in the testis, but have significantly reduced numbers of late meiotic spermatocytes and post-meiotic spermatids [41], have reduced Rep8 expression (Fig. 4C). Thus, the high levels of Rep8 expression in the testis depends on the presence of late meiotic spermatocytes/post-meiotic round spermatids in this tissue.

In order to test whether Rep8 is expressed in the developing germ cells in adult testes, we performed *in situ* hybridization. In adult mouse testes, high Rep8 expression was evident in the developing germ cells within the seminiferous tubules (Fig. 4D). Rep8 mRNA levels are low or undetectable in the interstitial somatic cells in the testis (Fig. 4D), in mitotic or meiotic male germ cells inside the testis tubules (Fig. 4E), and in early stages of post-meiotic round spermatid differentiation (Fig. 4E, stage I–VI). Rep8 mRNA is abundant during the late stages of round spermatid differentiation (Fig. 4E, stage VII–VIII), and as the round spermatids start to elongate (Fig. 4E, stage IX–X). However, Rep8 mRNA levels are low or undetectable in elongated spermatids (Fig. 4E, stage I–VI, stage VII–VIII). These *in situ* hybridization data are consistent with the qRT-PCR data for Rep8 expression in the testis. Thus, the high levels of Rep8 expression in the testis appear to be caused primarily by a pulse of Rep8 upregulation in germ cells during late round spermatid differentiation.

In order to test whether the high levels of Rep8 mRNA expression in the adult ovary [35] are also caused by expression in the developing germ cells, we performed *in situ* hybridization on adult ovaries. In contrast to the testis, the high levels of Rep8 expression in the ovary appear to be caused primarily by expression in the somatic cells rather than the germ cells (Fig. 4F). Rep8 mRNA levels are low or undetectable in the oocytes, and in the somatic corpora lutea and stromal cells present in the ovary (Fig. 4F). However, Rep8 mRNA is abundant in the somatic granulosa cells that surround the oocyte in the developing follicles (Fig. 4F). The high levels of Rep8 expression in ovaries and testes therefore does not appear to be caused by a common Rep8-expressing cell lineage in male and female gonads. Rather, Rep8 expression in adult gonads may be a consequence of some similarity between the cell biology of granulosa cells in the ovary and round spermatids in the testis.

### Rep8 associates with Hrd1

**Figure 5:**
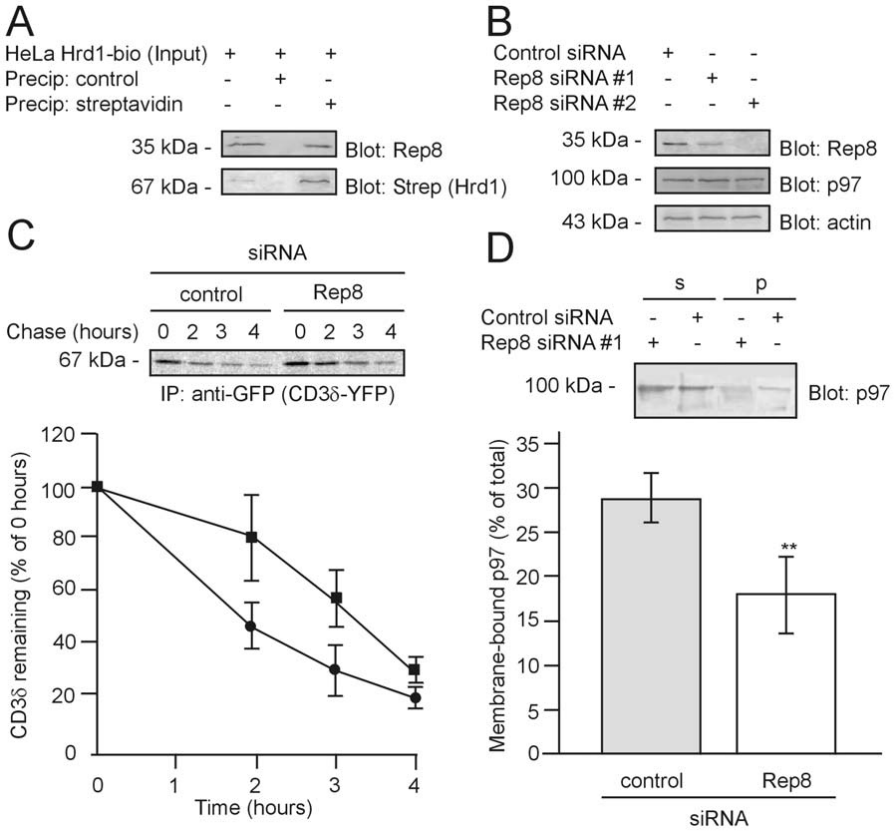
Rep8 binds Hrd1 and participates in ERAD. (A) HeLa cells expressing *in vivo* biotinylated Hrd1 were precipitated using streptavidin Sepharose beads, or as a control, IgG Sepharose beads, and analyzed by SDS-PAGE and blotting using the indicated probes. Rep8 was bound to the immobilized Hrd1. (B) Human MelJuSo cells were transfected with control siRNA or two different siRNAs specific for Rep8. After 5 days, the expression of the indicated proteins was analyzed by SDS-PAGE and blotting. Actin served as a loading control. (C) Pulse-chase experiments performed on cells expressing YFP-tagged CD3δ. The cells were transfected with Rep8 siRNA#2 (filled square) or control siRNA (filled circle). At the indicated times during the chase period the substrate was retrieved by precipitation using antibodies specific for GFP. The precipitated material was resolved by SDS-PAGE and visualized by phosphoimaging (top panel). Knock-down of Rep8 expression caused a decrease in the degradation rate (lower panel). (D) The amount of membrane-bound p97 in MelJuSo cells transfected with control siRNA or siRNA specific for Rep8 was determined by differential centrifugation and quantitative blotting. The error bars show the standard deviation. The difference was significant at the p<0.01 level (**) (t-test, n = 4).

Since we found that Rep8 is a cofactor of p97 that localizes to the ER membrane, we analyzed if Rep8 was associated with the E3 ubiquitin-protein ligase, Hrd1, that plays an important role in ERAD [8]. Extracts from HeLa cells, stably transfected to express biotin-tagged Hrd1, were precipitated with a streptavidin resin. Indeed, the biotinylated Hrd1 interacted with Rep8 (Fig. 5A). However, since we could not detect any interaction between recombinant Hrd1 and Rep8 purified from *E. coli* (not shown), the interaction observed in HeLa cells is probably indirect and bridged by p97 or other components of the ERAD system.

### Knock-down of Rep8 expression inhibits ERAD and p97-membrane interaction

Since Rep8 is an ER-membrane protein that interacts with p97, it is possible that proteolysis of ER proteins is impaired in cells with a decreased content of Rep8. To test this prediction, Rep8 expression in MelJuSo cells was knocked down with siRNA (Fig. 5B). Knock-down of Rep8 did not alter the level of p97 (Fig. 5B). Thus, cells do not compensate for the lack of Rep8 by inducing p97.

To test if Rep8 plays a role in ERAD, the degradation kinetics of the model ERAD substrates, TCRα and CD3δ, were analyzed. In both cases we observed a slight retardation in their degradation (Fig. 5C and Fig. S2). However, the degradation of a cytoplasmic p97-relevant proteasome substrate was unaffected (Fig. S3), indicating that Rep8 specifically targets ER-derived proteasome substrates.

We then speculated if Rep8 perhaps recruits p97 to the ER membrane. By differential centrifugation and quantitative immunoblotting, the amount of p97 associated with the microsome pellet was determined. In cells transfected with Rep8-specific siRNA, the amount of membrane-bound p97 was significantly reduced compared with the control (Fig. 5D). We therefore conclude that Rep8 tethers p97 to the ER membrane.

## Discussion

The ATPase complex, p97, is a molecular segregase [17] connected with a broad spectrum of cellular pathways, including fusion of ER and Golgi membranes [19], [45], DNA repair [46], transcription factor activation [18] and ERAD [11], [47]. The cellular mechanisms involved in directing p97 to these various functions are probably determined by its range of cofactors. Here, a previously cloned gene [35], encoding the uncharacterized human protein Rep8, was isolated as a p97 cofactor.

We found that Rep8 expression is highly tissue specific and most abundant in gonads. This is in accordance with previous results on the mRNA level [35]. Furthermore, we have shown that the high levels of Rep8 expression in the testis is caused by expression in the post-meiotic round spermatids within this tissue. Rep8 is not the only ERAD relevant protein known to reside in the testes. Previous studies have found testis-specific homologues of calnexin (Clgn) and protein disulfide isomerase (Pdilt) interact to form a testis-specific chaperone complex in post-meiotic germ cells [48]–[50]. Sperm from *Clgn^−/−^* mice are infertile and exhibit defects in the heterodimerization and maturation of egg-binding glycoproteins in the ER during spermatogenesis. These egg-binding proteins are not present on the surface of mature *Clgn^−/−^* sperm, and *Clgn^−/−^* sperm have defects in egg-binding at fertilization [51]–[53]. Interestingly, like Rep8, Clgn and Pdilt are highly expressed in round spermatids, a stage of spermatogenesis where cell-surface and secreted proteins, that will eventually facilitate interactions between the sperm and the egg during fertilization, are being synthesized. Perhaps the special pH, temperature and redox environment, or the presence of specific substrates in round spermatids may require expression of ERAD proteins such as Rep8. Indeed, components of the insoluble protein matrix that is present in the specialized acrosome structure, where many of these secreted proteins are stored, may be prone to aggregation and misfolding [54]. The generation of *Rep8^−/−^* knockout mice will be required to analyze the role of Rep8 in reproductive tissues in more detail. Although nematodes do not encode any apparent orthologues of Rep8, simultaneous knock-down of several UBX domain proteins results in a germ-line phenotype that does not produce sperm [55]. Perhaps mice lacking Rep8 will display a similar phenotype.

Rep8 contains a transmembrane domain, a VIMP-like region and a UBX domain. VIMP is also a transmembrane protein that localizes to the ER-membrane and plays a role in ERAD [29]. It is known that VIMP associates with p97 [29], but VIMP does not contain a UBX domain and the p97 interaction region in VIMP has yet to be identified and characterized. Another important difference between VIMP and Rep8 is that VIMP is most likely a selenoprotein whereas Rep8 is not. The region in VIMP which is homologous to Rep8 is located just next to the transmembrane domain. The structure of this region of VIMP is basically an extended helix [PDB: 2QF2], which probably functions as a spacer to keep the VIMP C-terminal area at a distance from the membrane. Presumably the VIMP-like area in Rep8 shares this function.

The UBX domain of Rep8 is sufficient and necessary for interacting with the p97 N-domain. We also found that Rep8 interacts with the E3 ubiquitin-protein ligase Hrd1. However, since we were unable to reconstitute this interaction using purified components *in vitro*, we speculate that this interaction is indirect and bridged by other components of the ERAD machinery, perhaps by p97 itself, which interacts with Hrd1 directly [56]–[58]. Since Rep8 and Hrd1 both associate with the p97 N-domain their interaction with p97 is probably mutually exclusive. However, due to the hexameric structure of p97, the ATPase may in principle associate simultaneously with up to six different N-domain binding partners, and ternary Rep8-p97-Hrd1 complexes could therefore be relevant. For some p97 cofactors, interaction critically depends on p97's prior association with other cofactors [59]. In the future it would be interesting to see if Rep8 and VIMP co-associate with p97.

On the subcellular level, Rep8, like VIMP [29], localizes to the ER-membrane. The Rep8 C-terminal UBX domain faces the cytoplasm and thus connects p97 to this organelle. Accordingly, when Rep8 is lacking less p97 is associated with the ER membrane. Conversely overexpression of Rep8 would presumably shift p97 to be more tightly connected with the ER membrane. However, unfortunately we were unable to overexpress Rep8 to a significant level.

We found that upon knock-down of Rep8 expression, proteolysis of two model ERAD substrates was slightly retarded. We note that p97 plays other important roles at the ER-membrane, including transcription factor maturation [18] and membrane fusion [45], and we cannot rule out that the observed effect on ERAD is indirect and perhaps due to a perturbed p97 cofactor binding in response to the reduced amount of Rep8. Alterations in p97 cofactor binding may cause pleiotropic phenotypes and has been linked to disease [60].

As mentioned, Hrd1 may itself bind p97 directly, and it is therefore surprising that other p97 binding partners are needed to fulfill the same function. However, besides Hrd1 and Rep8, other transmembrane ERAD components such as VIMP [29], Erasin [30], and Derlin-1 [58] also bind p97 directly. This redundancy may explain why cells lacking Rep8 only display the moderate ERAD phenotype described here. Given the mild effect of Rep8 on the ERAD model substrates used here, another possibility is that Rep8 is involved in degradation of clients specific for reproductive tissue.

## Supporting Information

Figure S1 **Rep8 is a phylogenetically conserved protein in higher eukaryotes.** Clustal W (v1.82) alignment of human (*Hs*) Rep8 with its bovine (*Bt*), mouse (*Mm*) and zebrafish (*Br*) orthologues. Identical and similar residues have been marked. The domain organization is indicated by the colored bars. Rep8 contains a signal sequence (red), a transmembrane domain (black), a region which is homologous to VIMP (blue), and a UBX domain (green).
(TIF)

Figure S2 **Degradation of TCRα.** Pulse-chase experiments performed on cells expressing HA-tagged TCRα. The cells were transfected with Rep8 siRNA#2 or control siRNA. At the indicated times during the chase period the substrate was retrieved by precipitation using antibodies specific for HA. The precipitated material was resolved by SDS-PAGE and visualized by phosphoimaging. Slower migrating species (filled arrow) corresponding to glycosylated forms of the protein were visible. Knockdown of Rep8 expression caused a decrease in the degradation.
(TIF)

Figure S3 **Rep8 does not affect degradation of a cytoplasmic proteasome substrate.** Pulse-chase experiments were performed on MelJuSo cells expressing ubiquitin-G76V-YFP transfected with Rep8 siRNA#2 or control siRNA. At the indicated times during the chase period, ubiquitin-G76V-YFP was precipitated using antibodies specific for GFP. The precipitated material was resolved by SDS-PAGE and visualized by phosphoimaging. The asterisks (*) marks an unknown contaminant.
(TIF)

Table S1 **Sequences of primers used for qRT-PCR.**
(PDF)
