# Supplementary figures and images for: The Tissue-Specific Rep8/UBXD6 Tethers p97 to the Endoplasmic Reticulum Membrane for Degradation of Misfolded Proteins
Source: PLoS One. 2011 Sep 15;6(9):e25061. doi: 10.1371/journal.pone.0025061 (PMC3174242; doi:10.1371/journal.pone.0025061)

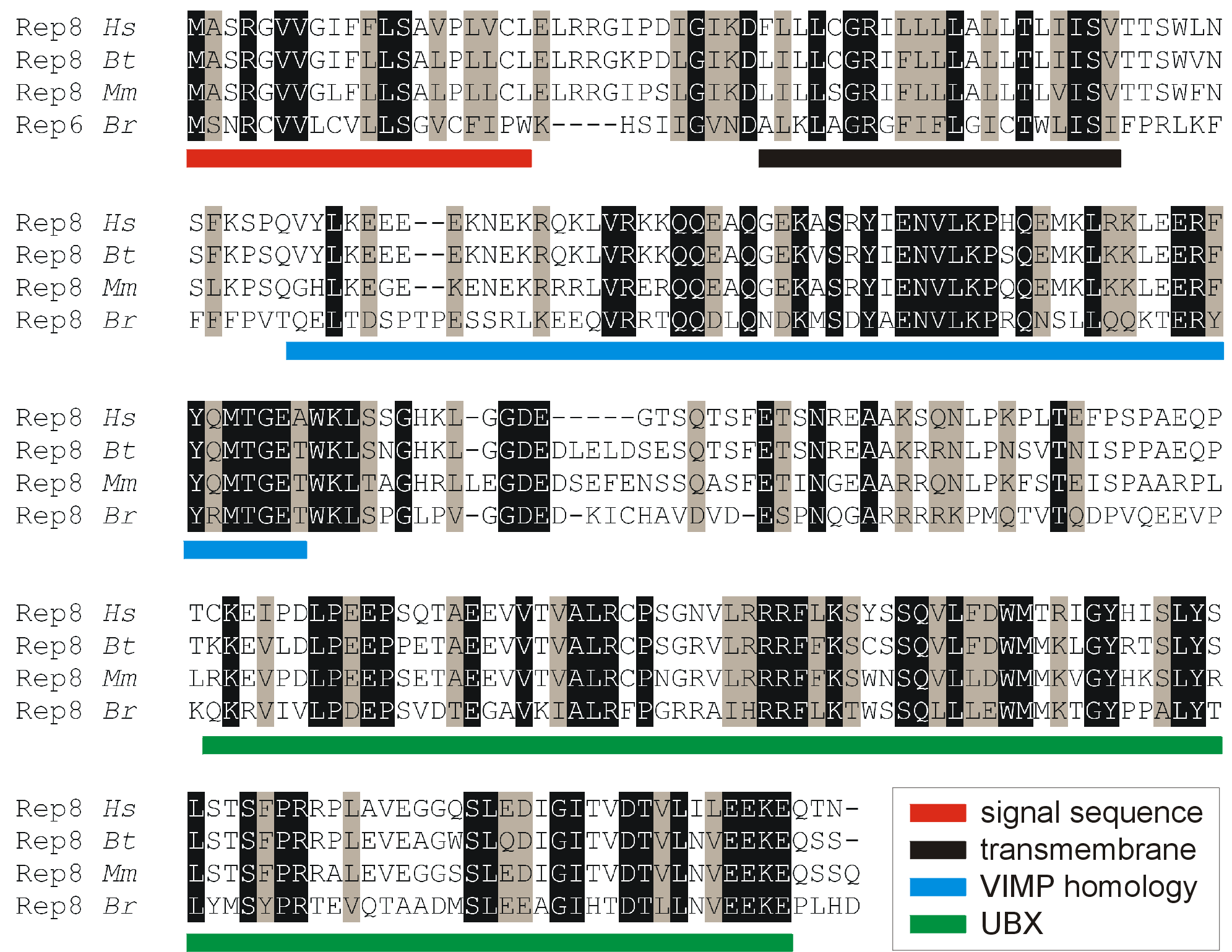

Supplement: Figure S1 — Rep8 is a phylogenetically conserved protein in higher eukaryotes. Clustal W (v1.82) alignment of human (Hs) Rep8 with its bovine (Bt), mouse (Mm) and zebrafish (Br) orthologues. Identical and similar residues have been marked. The domain organization is indicated by the colored bars. Rep8 contains a signal sequence (red), a transmembrane domain (black), a region which is homologous to VIMP (blue), and a UBX domain (green). (TIF) [file pone.0025061.s001.tif]

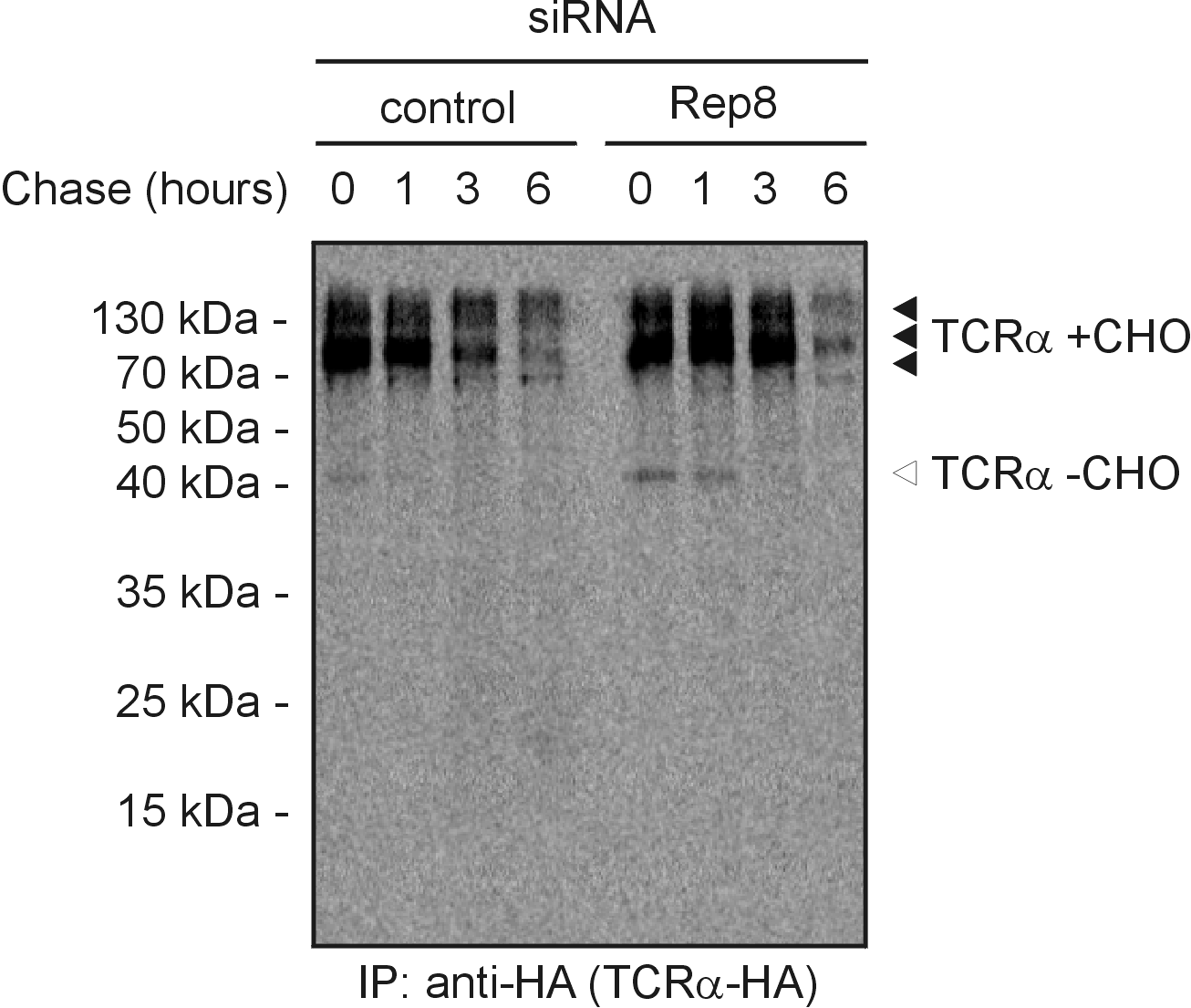

Supplement: Figure S2 — Degradation of TCRα. Pulse-chase experiments performed on cells expressing HA-tagged TCRα. The cells were transfected with Rep8 siRNA#2 or control siRNA. At the indicated times during the chase period the substrate was retrieved by precipitation using antibodies specific for HA. The precipitated material was resolved by SDS-PAGE and visualized by phosphoimaging. Slower migrating species (filled arrow) corresponding to glycosylated forms of the protein were visible. Knockdown of Rep8 expression caused a decrease in the degradation. (TIF) [file pone.0025061.s002.tif]

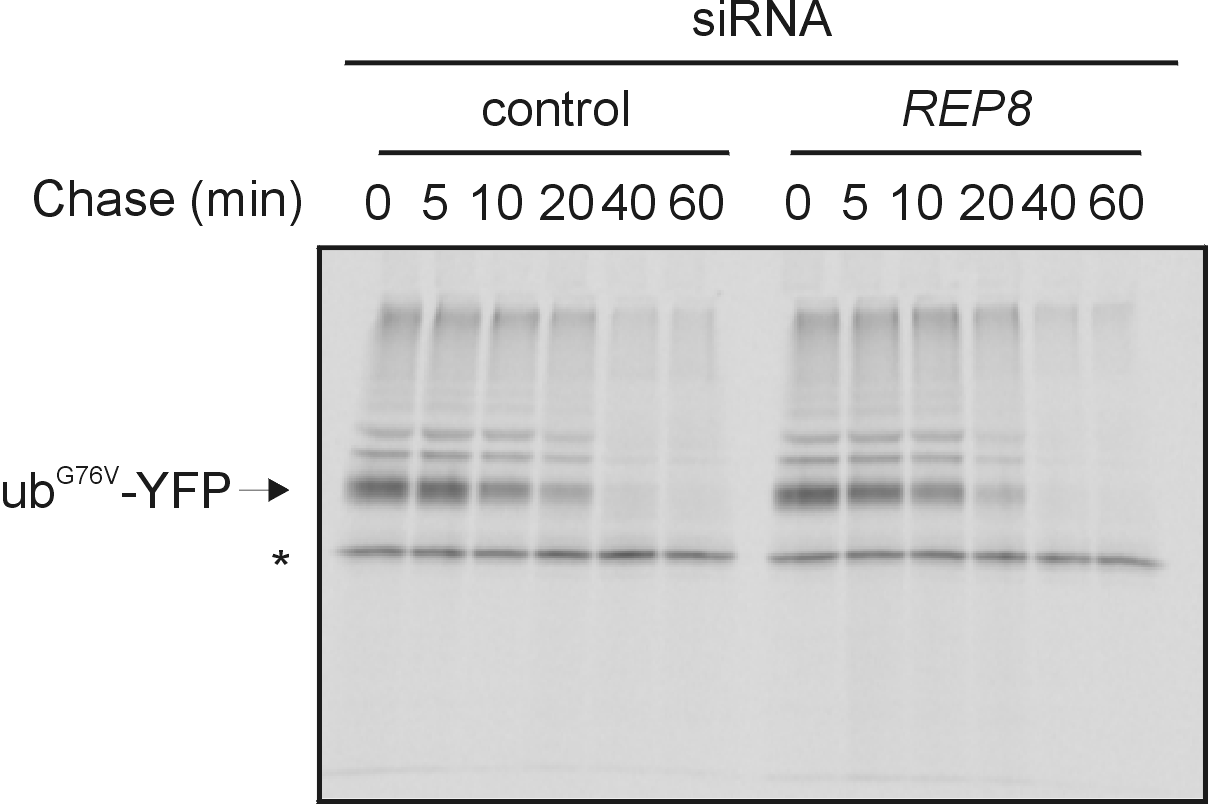

Supplement: Figure S3 — Rep8 does not affect degradation of a cytoplasmic proteasome substrate. Pulse-chase experiments were performed on MelJuSo cells expressing ubiquitin-G76V-YFP transfected with Rep8 siRNA#2 or control siRNA. At the indicated times during the chase period, ubiquitin-G76V-YFP was precipitated using antibodies specific for GFP. The precipitated material was resolved by SDS-PAGE and visualized by phosphoimaging. The asterisks (*) marks an unknown contaminant. (TIF) [file pone.0025061.s003.tif]
