# Supplementary material for: The Tissue-Specific Rep8/UBXD6 Tethers p97 to the Endoplasmic Reticulum Membrane for Degradation of Misfolded Proteins
Source: PLoS One. 2011 Sep 15;6(9):e25061. doi: 10.1371/journal.pone.0025061 (PMC3174242; doi:10.1371/journal.pone.0025061)
Supplement: Table S1 — Sequences of primers used for qRT-PCR. (PDF) [file pone.0025061.s004.pdf]

Table S1

Sequences of primers used for qRT-PCR

Gene	Primer Sequence
β-Actin	5′- GGCTGTATTCCCCTCCATCG -3′
	5′- ACATGGCATTGTTACCAACTGG -3′
Sdmg1	5′- TCTGTGTTGAGATGCTGTTCG -3′
	5′- AGGCTGGCGAGAAGTTATGA -3′
Stra8	5′- CTATGTTTGCCACCTGCAAC -3′
	5′- AAAGGTCTCCAGGCACTTCA -3′
Sycp3	5′- TGCTGCTGAGTTTCCATCAT -3′
	5′- GCAATGAAACAGATACACGAGC -3′
Prm1	5′- CCTTATGGTGTATGAGCGGC -3′
	5′- ACCATGGCCAGATACCGAT -3′
Rep8	5′- CAGATGACAGGGGAGACCTG -3′
	5′- TTGCTGCCTCTCCGTTTATT -3′
